# The use of novel selectivity metrics in kinase research

**DOI:** 10.1186/s12859-016-1413-y

**Published:** 2017-01-05

**Authors:** Nicolas Bosc, Christophe Meyer, Pascal Bonnet

**Affiliations:** 1 Institut de Chimie Organique et Analytique (ICOA), UMR CNRS-Université d’Orléans 7311, Université d’Orléans BP 6759, Orléans Cedex 2, 45067 France; 2 Janssen-Cilag, Centre de Recherche Pharma Janssen-Cilag, Campus de Maigremont–CS 10615, Chaussée du Vexin, Val de Reuil, 27106 France

**Keywords:** Selectivity, Protein kinase, Kinase inhibitor, Gini score, Entropy score, Partition index, Window score, Ranking score

## Abstract

**Background:**

Compound selectivity is an important issue when developing a new drug. In many instances, a lack of selectivity can translate to increased toxicity. Protein kinases are particularly concerned with this issue because they share high sequence and structural similarity. However, selectivity may be assessed early on using data generated from protein kinase profiling panels.

**Results:**

To guide lead optimization in drug discovery projects, we propose herein two new selectivity metrics, namely window score (WS) and ranking score (RS). These metrics can be applied to standard in vitro data–including intrinsic enzyme activity/affinity (Ki, IC_50_ or percentage of inhibition), cell-based potency (percentage of effect, EC_50_) or even kinetics data (Kd, Kon and Koff). They are both easy to compute and offer different viewpoints from which to consider compound selectivity.

**Conclusions:**

We performed a comparative analysis of their respective performance on several data sets against already published selectivity metrics and analyzed how they might influence compound selection. Our results showed that the two new metrics bring additional information to prioritize compound selection.

**Graphical Abstract:**

Two novel metrics were developed to better estimate selectivity of compounds screened on multiple proteins.
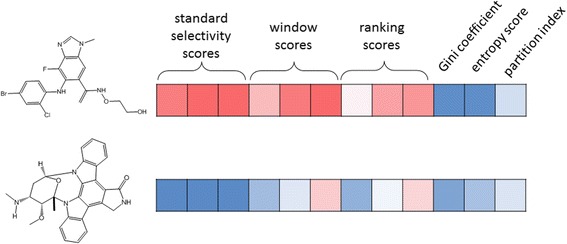

**Electronic supplementary material:**

The online version of this article (doi:10.1186/s12859-016-1413-y) contains supplementary material, which is available to authorized users.

## Background

The assessment of compound selectivity is of major interest in drug discovery. This important parameter is carefully monitored in drug discovery projects in order to minimize the potential toxicity liabilities of compounds. Selectivity assessment is often used in the definition of the Target Product Profile (TPP) of a lead to highlight the need for an acceptable window between activity on the biological target and off-target activities. An insufficient selectivity profile is one of the reasons underlying numerous failures in clinical trials [1]. Research in the field of protein kinase inhibitors is particularly impacted by this problem. The protein kinase family represents more than 500 members of the human proteome [2]. Due to the fact that they all share a well conserved ATP binding site, the design of selective inhibitors remains a challenge and has negatively impacted the progression of drug candidates to late-stage clinical development. The recent failures in early clinical trials of p38 MAPK inhibitors [3], as well as the difficulty of a CDK inhibitor (dinaciclib, PD-0332991) to reach phase III clinical trial [4] are noteworthy examples of the selectivity issues in the kinase field. Nonetheless, several studies have also shown that there are benefits to having selective but not necessarily completely specific inhibitors as drugs [5–7]. The success of multi-targeted kinase inhibitors imatinib and gefitinib shows that the safety of a drug does not rely exclusively on selectivity, as long as the administered dose remains within the therapeutic window [8–10]. Furthermore, a relative selectivity could be of added value to a compound, bringing with it a potentially advantageous polypharmacological profile [11]. For instance, dual inhibitors of the PI3K/mTOR pathway have been found to be more efficient at inhibiting the proliferation of breast cancer cells than specific inhibitors of PI3K or mTOR [12, 13]. This demonstrates the importance of studying the selectivity profile of compounds as soon as possible in the discovery process.

Historically, attempts to evaluate protein kinase inhibitor potency were only made on a reduced set of selected kinases. Thanks to recent improvements in biochemical assays, now there are a number of companies that offer robust and reliable technologies for screening compound collections on large kinase panels [14, 15]. Consequently, a growing number of studies have been published describing broad kinase profiling, in which tens to hundreds of compounds were tested on more than half of the human kinome [16–19]. These have made available to the scientific community a wealth of experimental data: such as binding affinity or inhibition of functional activity for the primary kinase target as well as the selectivity profile of the compounds screened. However, how the selectivity is defined or computed may highly influence the perception of molecules of interest. In the past decades, several selectivity metrics have been published and used for research purposes [20–22]. Since they differ significantly in their definition and complexity, this impacts both their ease of understanding and usability as well as the conclusions regarding compound selectivity profiles.

In this study, we propose two new metrics, named window score (WS) and ranking score (RS), to assess compound selectivity and we compare their performance on large kinase profiling data with four common selectivity scores commonly used in drug discovery: the standard selectivity score, the Gini coefficient, the selectivity entropy and the partition index. In this study, comparative assessment of the selectivity scores has been performed on three different published datasets obtained from different assay types. Two datasets were obtained from enzymatic assays performed on a large fraction of the kinome. For the third one, we used the results from a cellular assay to assess the performance of these selectivity metrics. The proposed metrics provide novel insights on the selectivity profile of tested molecules and can be used in drug discovery projects as decision making tools to help compound selection.

## Methods

### Datasets

Among all publicly available biological datasets containing protein kinases, we have selected the two datasets having the highest percentage of completeness, i.e. the interaction matrix is as complete as possible. Such a selection is particularly important to ensure a reliable comparison between various selectivity metrics. The first dataset was provided by Davis et al. [16], in which 72 inhibitors were tested on 442 wild-type and mutated protein kinases (completeness of 100%). A dissociation constant (Kd) was measured for each protein-ligand pair only when an activity was first detected at 10 μM compound concentration. Among the tested protein kinases, we removed three bacterial proteins leading to a total of 439 protein kinases. The second dataset was published by Anastassiadis et al. [17], where the percentage inhibition of 178 compounds, tested at 0.5 μM, were measured on 300 wild-type and mutated protein kinases (completeness of 99%).

The use of a unique metric to estimate the selectivity of a compound across different datasets, regardless of the experimental method used to generate these data would be of great value. Indeed it would ease data analysis by avoiding activity unit conversions, and it would provide a universal tool allowing the direct comparison of different selectivity profiles. Although the experimental screening techniques used in both datasets are different (competition binding assay [23] and “HotSpot” assay [17]), we observed a relatively important overlap between the protein kinase–ligand pairs tested. This is why these two datasets were used for direct comparison of various selectivity scores. Therefore, we standardized protein kinase names using the UniProt [24] identifier and compound names using InChI [25] identifier in both datasets for direct comparisons. We counted 4720 pairs present in both publications (249 protein kinases and 19 compounds). For convenience, we will refer to the Davis et al., and Anastassiadis et al. datasets, as Ambit and Reaction Biology datasets respectively.

While most of the selectivity screening assays are performed on recombinant enzymes in biochemical assays, cellular assays can provide complementary information such as signaling pathways, cellular potency and cell permeation [26]. Moreover, recent studies have shown that assessment of compound selectivity using cellular or enzymatic assays may provide different results [27]. In order to evaluate the broad applicability of our approach, we have also studied the different selectivity scores on cellular potencies. The NCI-60 Developmental Therapeutics Program Human Tumor Cell Line Screen was initiated in the late 1980s. A total of 60 cell lines were assembled to represent nine tumor types: breast, central nervous system, colon, leukemia, lung, melanoma, ovarian, prostate and renal [28]. Thousands of compounds have been screened on this panel to estimate their potency. Twenty-five protein kinase inhibitors approved by the Food and Drug Administration (FDA) or in clinical trials have already been tested on these cell lines. We collected these data using CellMiner^TM^, a web application developed by the Genomics and Bioinformatics Group of the NCI [29, 30]. We will refer this dataset as NCI-60 in the following text.

### Selectivity metrics

#### Standard selectivity score (S)

For a given compound, the standard selectivity score *S*(*x*), where *x* represents the activity threshold, is calculated by dividing the number of inhibited protein kinases having an experimental value greater than *x*, by the total number of tested protein kinases, as shown in the Eq. (1).

1 $$ S(x) = \frac{number\  of\  values\ge x\ }{total\  number\  of\  values} $$

Depending on the experimental method used in the assay, *x* may also express a logarithmic affinity or activity value. This metric is quantitative, easily computable and comparable between different profiling results. In this study, we have evaluated the variation of the standard selectivity score, *S*(*x*), by using three different thresholds. For the Ambit dataset we calculated S(pKd5), S(pKd6) and S(pKd7) for 5, 6 and 7 logarithmic Kd thresholds respectively. For the Reaction Biology dataset, we selected similar range of activities S(50%), S(70%) and S(80%) for 50, 70 and 80% of inhibition relative to control thresholds respectively. Finally for the NCI-60 data, we used the same threshold as for the Ambit dataset. Hence, we calculated S(pIC_50_5), S(pIC_50_6) and S(pIC_50_7). In each example, a low value of *S*(*x*) represents a high compound selectivity whereas a high value of *S*(*x*) reflects a poor selectivity. The thresholds were chosen to depict respectively low, medium and high biological interaction with protein kinases.

The main drawback of this metric is the applied threshold which needs to be defined and which has an important impact on the final result. For instance, we can represent the results of a profiling panel in which we take a compound tested on 100 protein kinases. We calculate the standard selectivity score S(pKd6) corresponding to a threshold of six for pKd. If we count three proteins with an affinity above the threshold, we may have apparently a selective compound (three inhibited proteins out of 100). Indeed, the S(pKd6) score would be 0.03. However this selectivity score does not give any information on the strength of binding of the compound and we would suppose that the three inhibited proteins will have similar effects on cells. In contrast, with the same conditions and three hits, we may have the situation where the ligand shows nanomolar affinity for one target and just above the threshold for another two. In this case, we would obtain the same standard selectivity score, however this profile is rather different. Whilst the compound binds all three proteins selectively over the rest of the panel, there is a net difference in affinity for the targeted proteins, with a consequent divergence in impact at the cellular level.

**Fig. 1:**
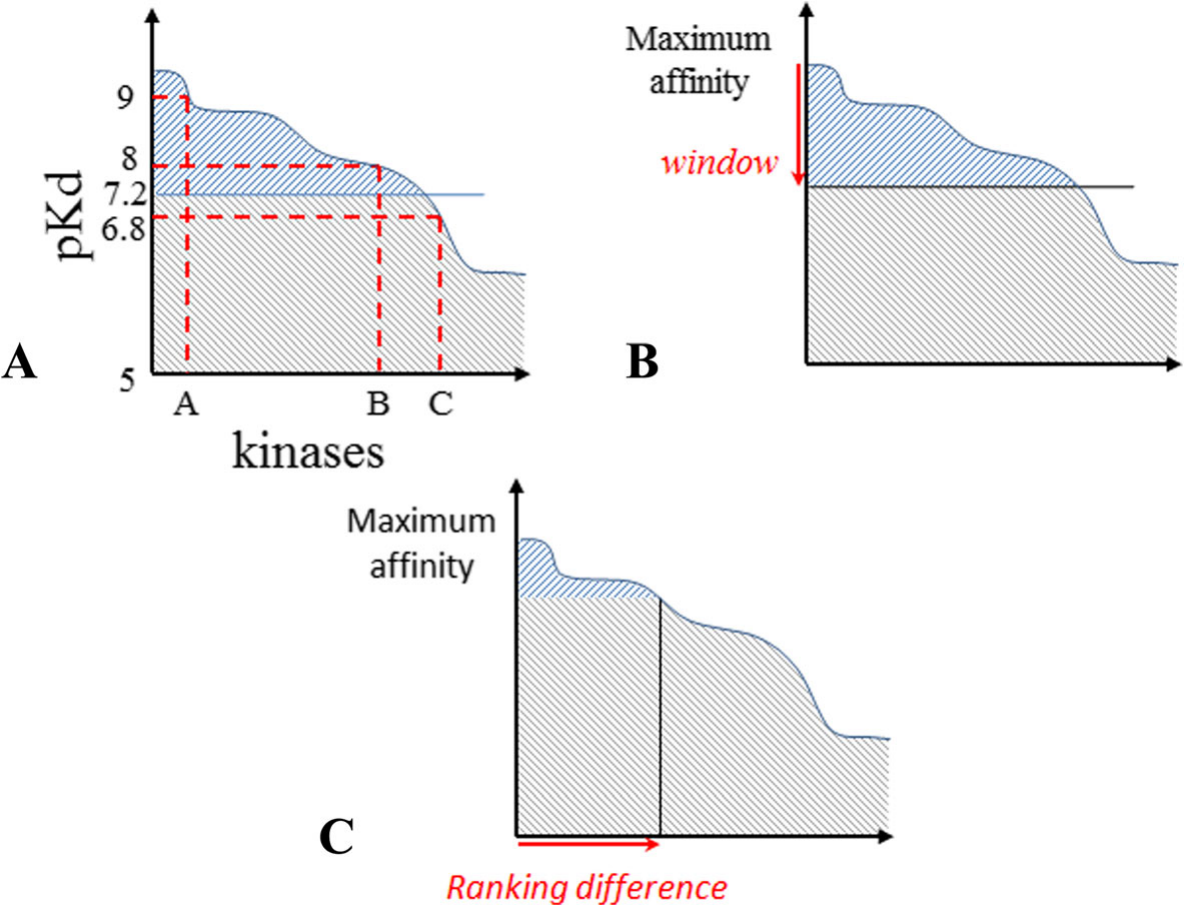
Schematic representation of three selectivity metrics. **a** standard selectivity score; **b** window score; **c** ranking score. The x-axis represents the protein kinases tested, the y-axis the activity of a given compound

Moreover, as recalled by Cheng et al. [21], the standard selectivity score does not capture any nuances. The use of the threshold will split the data into two categories and even if the values are clustered around the threshold, the protein kinases may well find themselves grouped into distinct categories. Figure 1a illustrates this specific issue where, in the proposed example, the pKd threshold of seven sets kinase B (pKd = 7.2) and kinase C (pKd = 6.8) in two different categories. While kinase A (pKd = 8) is in the same category as kinase B, though these two kinases have a larger affinity difference than kinase B and C. This is an issue frequently encountered when using a threshold for separating data and defining classes.

#### Gini coefficient (Gini)

The Gini selectivity metric (also known as the Gini coefficient) is particularly well designed for percentage of inhibition. According to Graczyk the data have to be first sorted in ascending order to generate the curve of the cumulative fraction of inhibition. The Gini coefficient is then calculated using the area under the curve. For further details of the calculation, the reader may refer to the original article [20]. Unlike the standard selectivity score, this metric does not rely on any threshold. However, the use of percentages of inhibition instead of a constant of inhibition (Ki) or a dissociation constant (Kd), makes the Gini coefficient highly dependent on the experimental conditions. Since the percentage of inhibition of a compound on a target is strongly dependent on its concentration, the Gini coefficient may vary at different compound concentrations.

#### Partition Index (PI)

The Partition Index (PI) was developed to discriminate selective compounds in small panels [21]. Using Kd values and a thermodynamic approach (a concept similar to the one applied for the selectivity entropy), this metric estimates the fraction of the binding of a compound onto a reference protein kinase in comparison to the remaining kinases in the panel. This metric returns partition index values P ranging from 0 to 1. A compound with a *P* value close to 1 would indicate a promiscuous compound that binds almost every protein. While this selectivity score is very useful within a unique drug discovery project, the use of a reference protein kinase to evaluate the partition index could be a limiting factor if one wants to compare selectivity scores between compounds from various projects having different primary targets. As mentioned by the authors, the partition index is mainly suitable for a hit-to-lead process where few selected proteins are used, but it may not be applicable for a larger protein panel. To override this issue, a P_MAX_ index was introduced to represent the inhibitor partitioning to the most potently inhibited protein kinase.

#### Selectivity entropy (Ssel)

The selectivity entropy (Ssel) is based on a thermodynamics approach to measure compound selectivity [22]. The authors assume that the system contains theoretically all protein targets from the assay panel without any competitive molecules such as ATP. Then the inhibitor is added in such a way that it does not saturate any target, but all inhibitor molecules bind a target. In such system, a selective inhibitor will bind approximately one specific protein, so will have low entropy, while a non-selective molecule will bind many targets and so represents high entropy. Applying thermodynamic principles, they translate this theory using the Boltzmann law and obtain a selectivity value based on the entropy of the system.

#### Window score (WS)

The following two novel metrics, respectively window score (WS) and ranking score (RS), require also a user-defined threshold. Nevertheless, they both meet three essential conditions. They take into account the ranking of the experimental biological data and can be calculated easily. Indeed, for a given compound, they rely on the distance separating its maximum affinity (or activity) amongst all the biological data from a user-defined affinity (or activity). Indeed, these calculated distances are important for analyzing the selectivity profile of a hit selected for hit-to-lead progression. Importantly, these two metrics can be applied on any data types such as affinity or activity data. In the current study, we applied the two selectivity scores on two datasets using pKd and percentage of inhibition data respectively, but they can also be used on other data types such as thermal stability shift assays [19].

For a given compound in a dataset, the window score requires the affinities to be ranked in descending order (from the highest pKd value to the lowest). By choosing a window threshold, we count all the affinity data points that are included in the interval between the highest pKd value and the highest pKd value minus the defined window (Fig. 1b). This number is then divided by the total number of tested proteins Eq. (2). Thus, the lower the window score, the more selective the compound.

2 $$ WS(x)=\frac{number\  of\; affinities\  in\  window(x)}{total\  number\  of\; affinities} $$

where window(x) represents the highest affinity minus a user-defined affinity. The window score ranges from almost zero (mathematically it cannot be lower than 1/total no. of affinity data points) to one. The higher the window score, the worse the selectivity. Similarly to the standard selectivity score mentioned above, the window score requires a threshold defined by the user. However, this threshold is not abstract because it defines the activity gap one may want to obtain between the most inhibited protein, often being the primary target, and the least inhibited protein in the given window. To evaluate the influence of the threshold on the window score, we chose three different windows: 2 log, 1 log and 0.5 log of Kd or IC_50_ (respectively annotated WS(pKd2), WS(pKd1), WS(pKd0.5) and WS(pIC_50_2), WS(pIC_50_1), WS(pIC_50_0.5)) for the Ambit and the NCI-60 datasets respectively, and 20, 10 and 5% of inhibition (respectively annotated WS(20%), WS(10%) and WS(5%)) for the Reaction Biology dataset.

#### Ranking score (RS)

For a given compound in a dataset, the ranking score (RS) requires the affinity values to be ordered from the highest to the lowest. By choosing a rank number, we subtract the affinity value at the position defined by the rank number from the best affinity Eq. (3) (Fig. 1c). Therefore, the higher the ranking score, the more selective is the compound.

3 $$ RS(x)= max(affinity) - affinity\  at\  rank\ x $$

where x represents the affinity rank. As seen in Eq. (3), a threshold also needs to be defined. The user only needs to define the rank corresponding to the activity difference between the activities of the xth inhibited protein and the most inhibited protein. In this study, we evaluate the influence of the threshold on the ranking score by selecting three different thresholds: 20, 10 and 5 (respectively RS(20), RS(10) and RS(5)) for the three datasets. Since this score uses a rank number it is independent of data types. Therefore, it can be applied for comparing compound selectivity profiles from a panel of proteins using various experimental techniques.

## Results

In this paper, we introduced two new metrics, namely window score (WS) and ranking score (RS), to assess the selectivity of compounds tested on a large panel of protein kinases and we compared the results with previously published selectivity scores. Comparative assessment of the selectivity scores has been performed on three different publicly available datasets. For each dataset, the calculation of each of the aforementioned metrics was performed with a Knime workflow (available upon request) and each molecule was then ranked according to each metric.

Since the Gini coefficient, the selectivity entropy and the partition index work exclusively with specific datatypes (percentages of inhibition for the Gini coefficient and Kd/Ki/IC_50_ for the two others) the calculation of all these metrics for each dataset required that we converted the data using an approximation of the Hill formula (4). As an example, Eq. (4) was used to convert percentage of inhibition to an approximate IC_50_:

4 $$ I{C}_{50}=\left[c\right]\left(\frac{100}{\%inh}-1\right) $$

where [c] is the compound concentration used for the screening and %inh is the measured percentage of inhibition.

### Results from the Ambit dataset

The Ambit dataset contains 72 protein kinase inhibitors tested on 441 protein kinases. For each pair, a quantitative dissociation constant (Kd) was measured if the primary affinity was below the compound concentration of 10 μM. For the compounds for which primary activity in the initial screen did not confirm, we assigned a value of 10.1 μM, considering the theoretical maximum affinity. This approximation allows us to have a data matrix of protein–ligand pairs with a degree of completeness of 100%. Thus, the selectivity scores calculated for all compounds are performed on the same number of protein kinases. To facilitate the comparison, all metrics have been ordered according to WS(pKd2) (Additional file 1: Table S1).

**Fig. 2:**
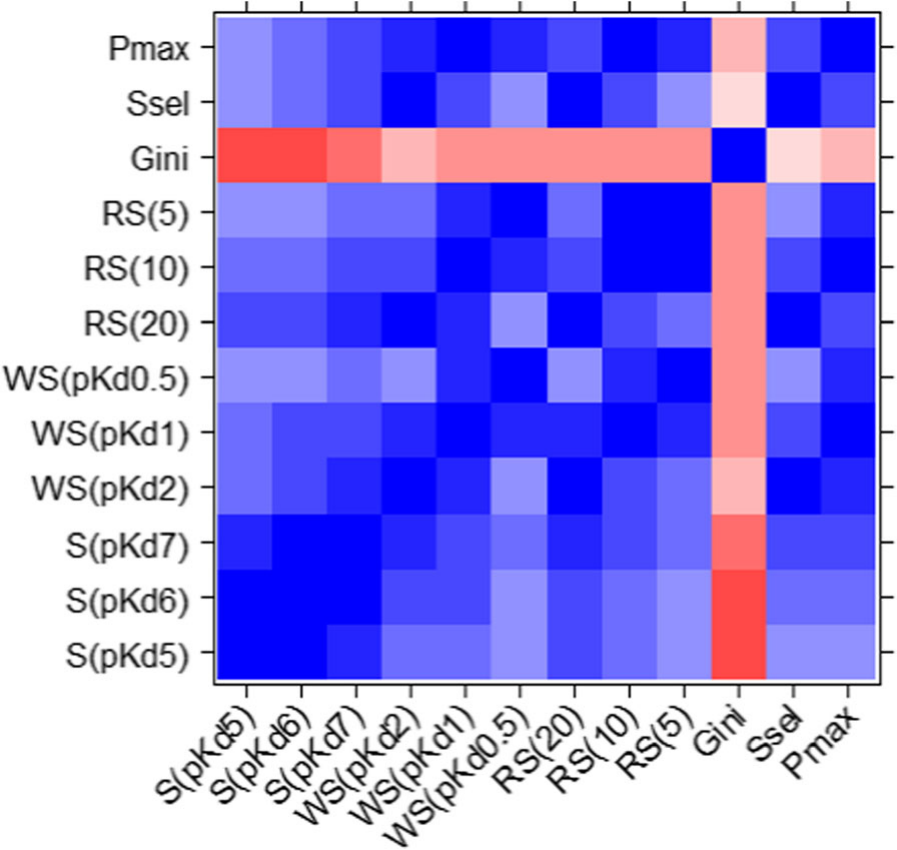
Ambit selectivity metric correlations. Values from −1 (*red*) to 1 (*blue*)

Depending on the applied threshold values, some selectivity scores cannot be computed. For instance, we noticed two compounds (CI-1040, BMS-345541) for which the standard selectivity score S(pKd7) could not be calculated because the threshold was too high. As a consequence, S(pKd7) was equal to zero since no protein kinase was inhibited above this threshold. This illustrates the concept that a compound may seem selective when the applied cut-off is too high. In other words, a compound may be inactive on protein kinases while the selectivity score could suggest the opposite. To avoid any confusion, we retained these compounds in the dataset but did not assign them a rank (Additional file 1: Table S1, empty cells). We compared the different metrics and observed that none of them offers the same exact selectivity rank. The standard selectivity scores (S(pKd5), S(pKd6) and S(pKd7)) present similar orders and it is confirmed by their good pairwise correlation *r* > 0.85 based on their respective ranks (Fig. 2).

In the top four molecules selected from each selectivity score S(pKd5) and S(pKd6), we retrieved the same four molecules namely GW-2580 [31], CI-1040 [32], MLN-120B [33] and SGX-523 [34]. S(pKd7) presents the same molecules at these positions except for CI-1040 because of the absence of strong affinity on the tested protein kinases. Indeed, despite a very high selectivity score with S(pKd6), this compound presents a maximum affinity pKd of 6.9 on MAP2K1 amongst all protein kinases. The correlation coefficient between S(pKd5) and S(pKd7) is 0.85, slightly lower than the ones observed between S(pKd5) and S(pKd6), and S(pKd6) and S(pKd7) (respectively 0.97 and 0.93, Fig. 2). This could be due to the identification of several equally selective compounds at high threshold value that prevent compound selectivity discrimination. The novel metrics (window score and ranking score) are also very sensitive to the threshold, but have the advantage of being based on a maximum affinity value. In that way, unlike the standard selectivity score, they cannot return a zero value even with a stringent threshold. We obtained similar ranking for WS(pKd2) and WS(pKd1) (Fig. 2, *r* = 0.73), and for WS(pKd1) and WS(pKd0.5) (Fig. 2, *r* = 0.75). However, a larger cut-off variation involves important differences in the rankings (Fig. 2, *r* = 0.37 between WS(pKd2) and WS(pKd0.5)). While BMS-387032/SNS-032 [35], nilotinib [36] and CHIR-265/RAF-265 are ranked respectively 14th, 24th and 28th by WS(pKd2), they are all ranked first according to WS(pKd0.5). Therefore, when the affinity rank x is high these three compounds have simultaneously many affinity values in the defined window. This is an advantage for this metric that allows a rapid identification of the affinity window between the best hit and the next one. We observed the same trend with the ranking score metric. The correlations between the ranking scores are high when the thresholds are similar while larger differences in the threshold led to low correlations (Fig. 2, *r* = 0.45 between RS(20) and RS(5), *r* = 0.64 between RS(20) and RS(10), *r* = 0.89 between RS(10) and RS(5)). Regarding the metric Ssel, which does not use any threshold, it presents a good correlation with several scores such as S(pKd7) (*r* = 0.63), WS(pKd2) (*r* = 0.90), RS(20) (*r* = 0.87) and P_MAX_ (*r* = 0.71). P_MAX_ is in good agreement with almost every other metric, in particular with WS(pKd2) (*r* = 0.74), WS(pKd1) (*r* = 0.86), WS(pKd0.5) (*r* = 0.79), RS(10) (*r* = 0.88), RS(5) (*r* = 0.84) and Ssel (*r* = 0.71). In general, all metrics used in this study seem to select the same molecules as being selective, and the same molecules as being unselective, with some specific differences in the ranking (Additional file 1: Table S1). Interestingly, only the Gini coefficient presents an anomalous ranking order with respect to the other metrics, characterized by several correlation coefficients below zero (Fig. 2).

When we analyzed the most selective compounds, we retrieved GSK-461364A [37] at the top position with the use of WS(pKd2), WS(pKd1), WS(pKd0.5), Ssel and P_MAX_. SGX-523 [34] is at the top position with WS(pKd0.5), RS(20), RS(10) and RS(5). PLX-4720 is ranked first by the three window scores. It is important to note that due to their mathematical form, some selectivity scores will provide an identical score for several molecules. MLN-120B is ranked first by S(pKd7), WS(pKd1) and WS(pKd0.5). AZD-6244/ARRY-886 is ranked first by S(pKd7), S(pKd6) and WS(pKd0.5). GW-2580 [31] is ranked first by S(pKd5), WS(pKd1) and WS(pKd0.5), though the global ranking of the compounds over each metric is quite different. VX-745 [38] is ranked first by S(pKd6), WS(pKd1) and WS(pKd0.5).

Some compounds show a good consensus among several metrics. Indeed, we found that GSK-461364A, PLX-4720, SGX-523, GDC-0879, BI-2536, CP-690550, GW-2580 and VX-745 are in the top ten for at least eight selectivity metrics. The selectivity entropy and the partition index contain the same top three molecules, and their top ten are in good agreement, but their rankings differ slightly for the remaining molecules (Fig. 2, *r* = 0.71).

We noticed that the use of a cut-off for some metrics can induce a lack of sensitivity for some molecules since the same score is obtained for several molecules. We observed this phenomenon mainly when a low cut-off value was applied for the standard selectivity or the window score. For instance, two and three compounds are ranked 13th and 14th respectively with S(pKd5). However, S(pKd6) identifies three compounds ranked first, two compounds ranked second and four compounds ranked 14th. Like the standard selectivity score, the window score can also suffer from this effect and 15 compounds are ranked first by WS(pKd0.5). Analyzing the affinities of these compounds, we noticed that their second best affinity is outside the 0.5 window and so, their respective window only contains one value, corresponding to the best affinity. However, although they have the same score, they do not bind the protein kinases with the same promiscuity. The maximum pKd for INCB18424 [39] is 10.4 on JAK2 kinase domain, while for AZD-6244/ARRY-886 [40] it is only seven on MAP2K1. Therefore, WS(pKd0.5) is well designed to identify compounds that bind preferentially to a protein kinase, but it is not suited to distinguish highly from compounds with poor activity.

**Fig. 3:**
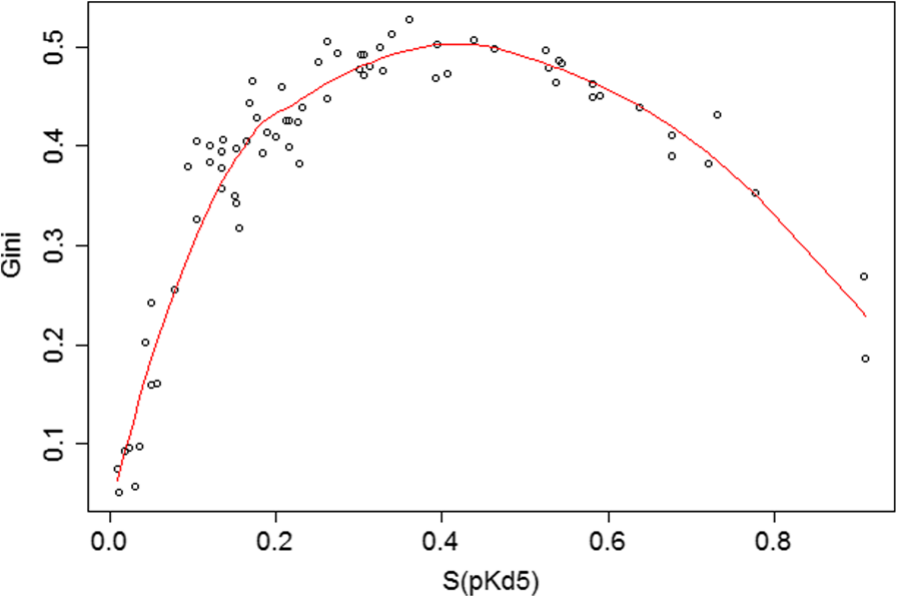
Correlation between the Gini coefficients and the standard selectivity score

Interestingly, the Gini coefficient is the metric providing the largest ranking differences compared to all other metrics (Fig. 2) with correlation values always close to 0 with the other metrics. As mentioned previously, this could be related to the conversion of Kd to percentage of inhibition, necessary to calculate the Gini coefficient [22]. When we plot the values of the S(pKd5) against the values of the Gini coefficients, we clearly see a parabolic relation that cannot be caught by a linear correlation (Fig. 3).

Surprisingly, we noticed that staurosporine, known to be a potent and unselective inhibitor of protein kinases [23], is ranked third with WS(pKd0.5) and 12th with WS(pKd1) (Additional file 1: Table S1) in contrast to the other metrics that instead rank this molecule as highly promiscuous. This clearly shows that the use of low thresholds for selectivity scores can lead to erroneous conclusions. In fact, staurosporine is ranked 47th, 55th and 61th by S(pKd7), S(pKd6) and S(pKd5) respectively. Finally, vandetanib, a FDA-approved drug for EGFR and VEGFR protein kinases, is classified as non-selective by all selectivity metrics.

### Results from the Reaction Biology dataset

The Reaction Biology dataset contains 178 protein kinase inhibitors tested on 300 protein kinases. Percentages of inhibition (instead of Kd) of each pair have been obtained at a single 0.5 μM compound concentration. Since the authors did not evaluate experimentally the inhibition profile for all protein-ligand pairs, the percentage of completeness is 99%. For the comparison between all metrics, they all have been ordered according to WS(10%) (Additional file 1: Table S2). As mentioned previously, we noticed that some compounds do not have activity greater than the thresholds of the standard selectivity scores. Therefore, we have calculated the twelve metrics on 109 out of the 178 compounds.

**Fig. 4:**
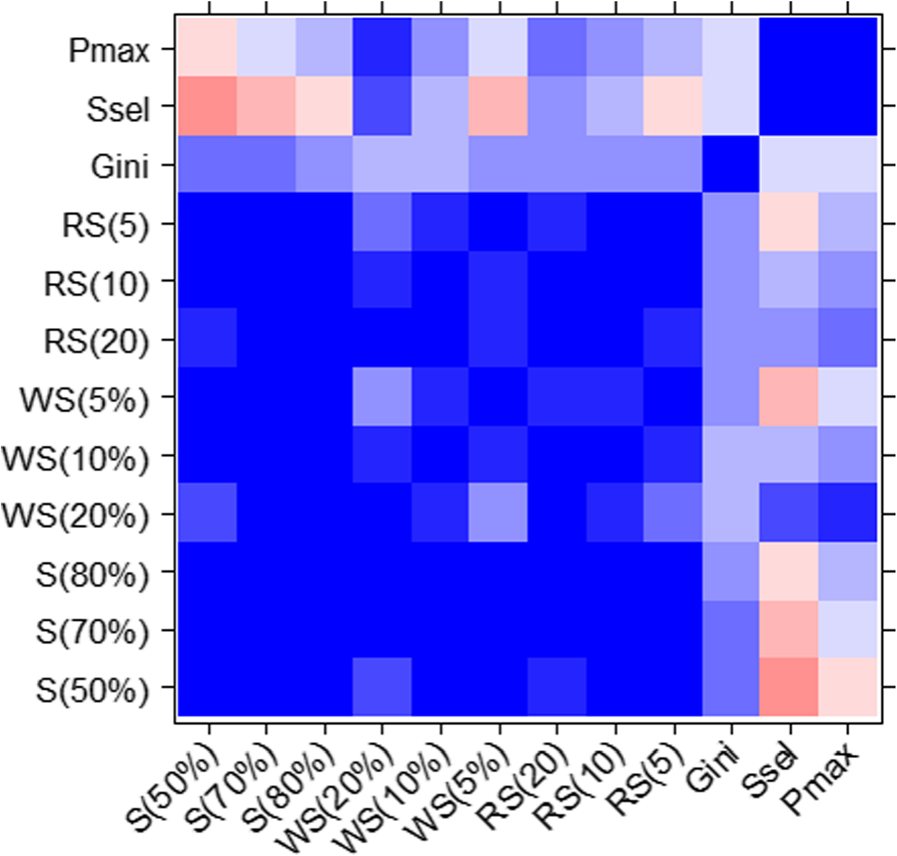
Reaction Biology selectivity metric correlations. Values from −1 (*red*) to 1 (*blue*)

The analysis of the results shows a global trend for the applied metrics. The color coding applied to Additional file 1: Table S2 clearly shows that the same molecules populate respectively the same ranking positions at the top and bottom parts of the table for almost all metrics except for the Gini coefficient, the selectivity entropy and the partition index. Figure 4 shows good pairwise correlations (*r* > 0.7) between the standard selectivity, the window and the ranking scores. The Gini coefficient shows low correlations with the other metrics though they are still higher than with the previous dataset. Hence, it seems that the Gini coefficient always ranks compounds differently compared to other metrics independent of the biological data type. PD 174265 is found as the most selective compound by S(70%), S(80%), WS(10%) and WS(5%). Moreover, this compound is ranked in the top ten by four other metrics (S(50%), WS(20%), RS(10) and RS(5)), and in the top 20 by three other metrics (RS(20), Ssel and P_MAX_). Surprisingly, this compound is ranked 131st by the Gini coefficient. The compound mentioned in the article as an EGFR inhibitor, presents a good consensus over the metrics. It is systematically ranked in the top ten of the tested compounds by all the metrics apart from P_MAX_ (11th) and Ssel (12th). Notably, it is ranked first by WS(20%) as well as all the RS metrics independently of the used thresholds. p38 MAP Kinase Inhibitor III seems also rather selective as it is ordered first by S(70%), S(80%), WS(10%) and WS(5%), and it appears in the top 20 for S(50%), WS(20%), RS(20), RS(10). Similarly, tandutinib is ranked first by S(70%), S(80%), WS(10%) and WS(5%) and in the top 20 by WS(20%), RS(20), RS(10) and Gini score. We retrieved the same first nine molecules using the P_MAX_ and Ssel metrics with almost identical order, but these compounds are far from being the most selective according to other metrics. As mentioned earlier, we converted the percentages of inhibition into IC_50_ values to calculate these two metrics. These transformed values may be responsible for such important differences. The Gini coefficient results in considerable ranking differences, as already reported [22].

Importantly, most of the metrics return the same set of compounds as being promiscuous. Sunitinib [41], SU11652 [42], JAK3 Inhibitor VI, GW 6976 [43], Indirubin Derivative E804, PKR Inhibitor [44], CDK1/2 Inhibitor II [45], SB 2180778 [46], K-252a [47] and staurosporine are always ranked amongst the least selective compounds by all metrics, except Ssel and P_MAX_. This suggests that, in general, the metrics employing a threshold show strong similarities in ranking compounds as suggested by their correlation coefficients. This conclusion differs slightly from what was observed with the Ambit dataset. This may be due to the use of percentage of inhibition which does not fully discriminate inactive, weakly active and highly active compounds compared to pKd or pIC_50_. Therefore, the effect of the threshold used with percentage of inhibition (5, 10, 20, 50, 70 and 80%) has a lower impact in discriminating selective compounds than pKd (0.5, 1, 2, 5, 6 and 7). However, in both examples, the Gini coefficient presents singular rankings. Since the Gini coefficient is calculated using cumulative inhibition of the compounds on the tested proteins, it is particularly sensitive to compound concentration and to the number of proteins screened.

## Discussion

### Selectivity metrics analysis of shared protein-ligand pairs

4720 protein-ligand pairs shared by Ambit and Reaction Biology datasets were retrieved. This represents 19 molecules and 249 protein kinases. As a result of the aforementioned threshold issue with the standard selectivity scores, we could not calculate the selectivity of some compounds using these metrics. The different rankings according to the metrics and the datasets are available in Additional file 1: Table S3.

Although we were interested in studying the impact of the nature of the activity data used to rank compounds, a direct consequence of the limited number of molecules (19) for which datasets they were available, was the over-sensitivity of the ranking to small variations in the data when comparing two metrics. Initially, we focused on the 19 molecules obtained from the Ambit dataset. Subsequently, we applied similar analyses using only the Reaction Biology dataset. Finally, we performed a pairwise comparison of each metric and analyzed the influence of the datasets. For the comparison we had to differentiate the metrics that rely directly (Gini score, selectivity entropy, partition index) or indirectly (standard selectivity score, window score) on the type of measured activity. Importantly, only the ranking score does not rely on activity type.

**Fig. 5:**
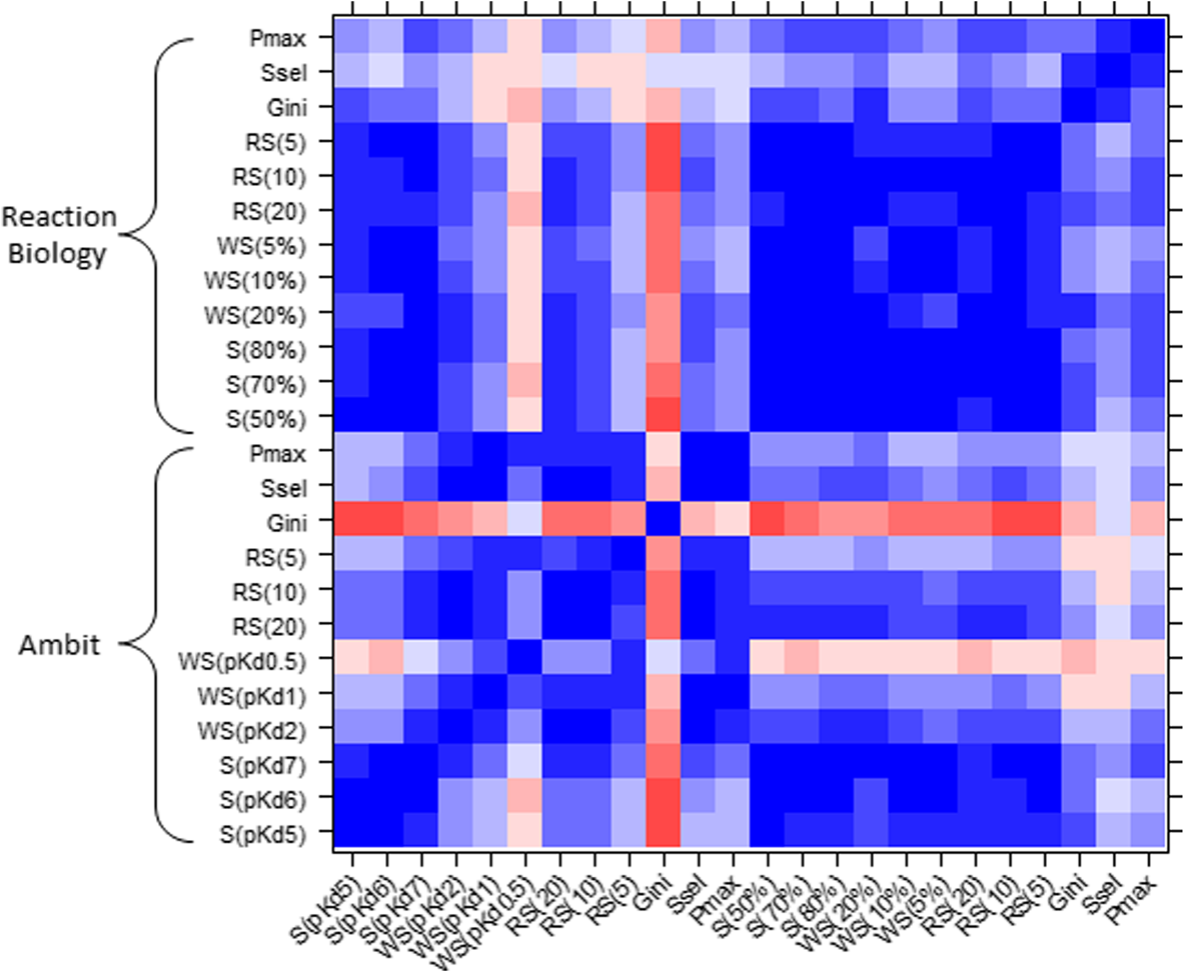
Ambit–Reaction Biology overlap metrics correlations. Values from −1 (*red*) to 1 (*blue*)

The results obtained using Ambit data confirmed the previous observations made on the full molecule set. S(pKd5) S(pKd6) and S(pKd7) all present similar ranking (Additional file 1: Table S3A) with pairwise correlations of at least 0.83 (Fig. 5). We also noticed similar rankings between WS(pKd2), RS(20), RS(10) and Ssel with good pairwise correlations (*r* > 0.85). Additionally, Ssel presents excellent correlations with WS(pKd1) (*r* = 0.91) and P_MAX_ (*r* = 0.91).

Gefitinib is ranked first in six cases (S(pKd7), WS(pKd2), WS(pKd1), WS(pKd0.5), Ssel and P_MAX_) and always in the top five for all other metrics. The only exception is with the Gini coefficient that ranks this molecule 10th. Lapatinib [48] and GW-2580 are also ranked systematically in the top five by the different metrics, except by the Gini coefficient that ranks the two compounds 19th and 18th respectively. Erlotinib appears rather selective in this panel since it is in the top five for nine out of the 12 metrics [49]. A consensus appears for staurosporine which is identified unselective by all calculated metrics. Finally, SKI-606 has an interesting profile since it is considered more or less selective depending on the metrics employed [50]. Indeed, with a pKd affinity greater than eight on 45 out of 249 tested protein kinases, the three standard selectivity scores rank this molecule among the least selective compounds. Contrastingly, WS(0.5) ranks SKI-606 first because there is only one activity present in the corresponding window. More precisely, it shows a very high affinity on ABL1 (pKd = 10.2) and the second highest affinity is greater than the 0.5 log threshold (pKd = 9.3 on MPK4K5). This compound is also well ranked by WS(pKd1) (third), RS(5) (6th), Gini (4th), Ssel (6th) and P_MAX_ (4th). Hence, SKI-606 clearly shows that the use of a selectivity score is subjective and depends on the applied metrics. The use of two different metric types such as RS or WS and Gini, selectivity entropy or partition index, provides greater confidence in the selectivity of a compound.

Regarding the 19 compounds from Reaction Biology data, we observed a similar tendency with all the compounds of this dataset (Additional file 1: Table S3B). Except with the Gini coefficient, the selectivity entropy and the partition index, all pairwise metrics correlate very well (Fig. 5). We retrieved gefitinib, erlotinib, MLN-518 [51], SB-203580 [52], imatinib [53] and GW-2580, almost every time in the top five by the standard selectivity score and the window score. AB-1010 is ranked first by the Gini coefficient, Ssel and P_MAX_ but other metrics rank this compound with a medium selectivity score [54]. Standard selectivity, window and ranking scores are in agreement in considering staurosporine, dasitinib [55] and SKI-606 as poorly selective. Notably, Gini coefficient, Ssel and P_MAX_ consider the PI3K inhibitor, PI-103 [56], as a promiscuous compound. PI-103 is a special case since it has been designed to target an atypical family of the kinome not present in the published dataset. Therefore, it inhibits weakly most of the typical protein kinases of the panel (no activity greater than 50%). However, WS(10%) and specially WS(5%) rank this compound as particularly selective since an important activity gap is observed between the first and second most inhibited kinases (CSNK2A2 at 44% and NUAK2 at 25%). Interestingly, SKI-606, which was well ranked by WS(pKd1) and WS(pKd0.5) on Ambit dataset, is ranked last by the two equivalent metrics WS(10%) and WS(5%) on Reaction Biology dataset .

Finally, to determine if one of these metrics could return approximately a similar rank regardless of the dataset and the measured activity type, we performed pairwise comparison of the compound ranks given by each metric (Additional file 1: Table S3A and B). S(pKd5) and S(50%) show a high correlation (*r* = 0.92), as do S(pKd6) and S(70%) (*r* = 0.90) and S(pKd7) and S(80%) (*r* = 0.91) (Fig. 5). Additionally, S(pKd7), computed on the Ambit dataset, correlates with the standard selectivity scores, the window scores and the ranking scores calculated on the Reaction Biology dataset. S(pKd6) and S(pKd7) correlate better with the window scores and the ranking scores calculated on the Reaction biology dataset than with those calculated on the Ambit dataset probably because the compounds have been tested at 0.5 μM, and a 50% inhibition of a compound roughly corresponds to a pIC_50_ (or pKd) of 6.3. The window scores calculated on the Ambit dataset differ slightly from those calculated on the Reaction Biology dataset. When the threshold decreases for the window score, the correlation between the two datasets decreases also. For instance, the correlation between WS(pKd2) and WS(50%) is equal to 0.75, but it decreases to 0.46 between WS(pKd1) and WS(20%), and drops to zero between WS(pKd0.5) and WS(10%) (Fig. 5).

Here again, this might be due to the correlation between pKd and percentage of inhibition ranges and a 2-log, 1-log and 0.5-log gaps correspond to 50, 20 and 10% activity gaps respectively. Regarding the ranking scores, we noted some interesting correlations between the two datasets using RS(20) (*r* = 0.74) and RS(10) (*r* = 0.71). These medium correlations clearly show significant differences in the selectivity results of the ranked compounds when two types of experimental data are used. Several outliers have also been identified [57]. A thorough analysis on each compound reveals that the major differences come from the highest activities, as reflected by the low correlation of the RS(5) (*r* = 0.34) between the two datasets. Finally, as seen previously, there is no correlation between the Gini coefficients calculated (*r* = −0.15), the Ssel (*r* = 0.13) or the PMAX (*r* = 0.25) probably due to activity data conversion necessary for these metrics (see Methods). In conclusion, none of these three metrics returns the same compound ranking when measured activities are provided by different experimental techniques. Nevertheless, the standard selectivity score and the window scores returned similar ranking for the 19 molecules studied. It would be interesting to carry out this comparison on larger datasets to confirm this trend [58].

### Selectivity analysis of kinase inhibitors from cellular screening data

We evaluated the twelve metrics on cellular potencies obtained from the US National Cancer Institute (NCI) panel [28]. We retrieved 25 protein kinase inhibitors tested on up to 60 human cancer cell lines. To avoid any bias, we removed the inhibitor lestaurtinib since it was tested on only 40 cell lines, and the melanoma cell line MDA_N used by only two inhibitors. For each metric, we ranked the 24 remaining protein kinase inhibitors (Additional file 1: Table S4).

Firstly, each metric offers a unique rank. Imatinib was ranked first or second by all metric except by the Gini coefficient (6th). Nilotinib also presents a good selectivity on these cell lines [36]. It is present in the top three of all metrics with the exception of S(pIC_50_5) and Gini. The results for bosutinib [50] and sunitib [42] show greater contrast. These two compounds are scored second and first by S(pIC_50_7), WS(pIC_50_1) and WS(pIC_50_0.5), fourth and third by RS(5), third and fourth by P_MAX_, but 14th and 13th by S(pIC_50_5), 14th and 17th by Gini, and 10th and 12th by Ssel respectively. Regarding the compounds with the lowest selectivity scores, sorafenib [59] and trametinib seem to be the least selective kinase inhibitors amongst the 24 kinase inhibitors [60, 61]. It is important to note that the MEK inhibitor trametinib is highly selective on the in-vitro biochemical kinase panel [60]. Interestingly, sorafenib is a drug approved by the FDA for the treatment of renal cell and hepatocellular carcinoma and also for locally recurrent metastatic, progressive differentiated thyroid carcinoma. Nevertheless, in these assays, sorafenib inhibits the 59 tumor cell lines at less than 10 μM, but its maximum inhibition is relatively high (1.25 μM on the breast cancer cell line MDA_MB_231), the most sensitive cell line.

**Fig. 6:**
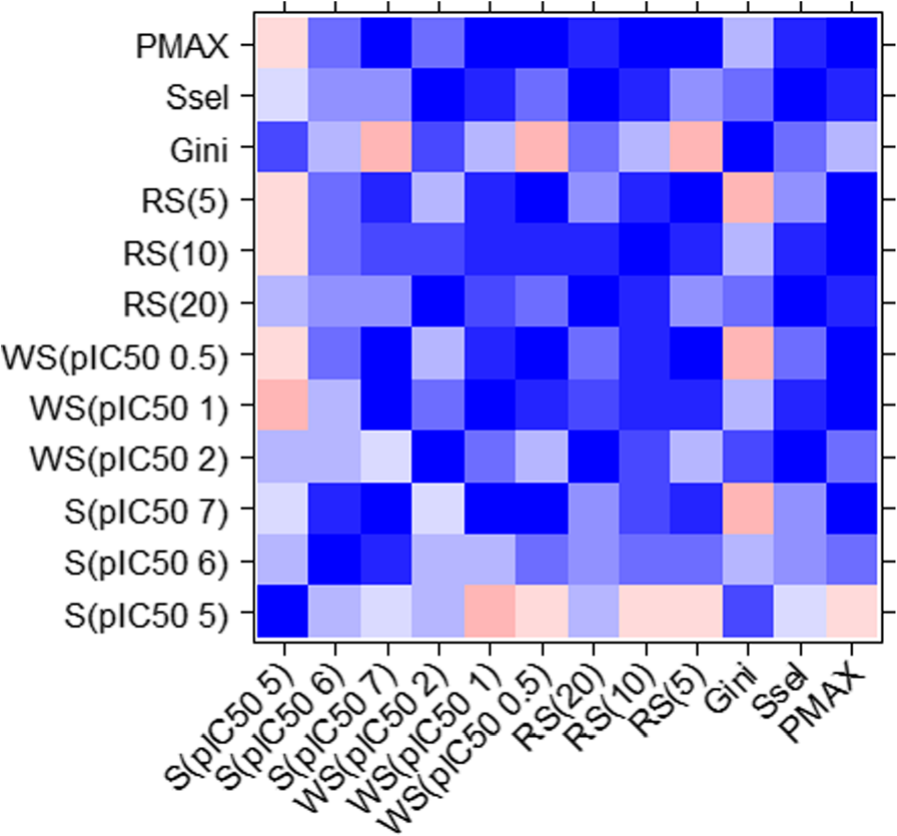
NCI-60 selectivity metric correlations. Values goes from −1 (*red*) to 1 (*blue*)

The correlation matrix allows the detection of novel patterns regarding the differences in selectivity metrics (Fig. 6). Firstly, the standard selectivity score S(pIC_50_5) poorly correlates with most of the metrics, even with the S(pIC_50_6) (*r* = 0.27) and S(pIC_50_7) (*r* = 0). In contrast, S(pIC_50_7) shows a high correlation with most of the other metrics, because these correlations were calculated taking into account only compounds for which S(pIC_50_7) could be estimated (17 out of 24 compounds). This is due to the reduced number of compounds with high cell-based potency compared to those observed with high activity in enzymatic assays. WS(pIC_50_2) does not correlate with any of the other window scores (*r* = 0.54 with WS(pIC_50_1) and *r* = 0.23 with WS(pIC_50_0.5)) due to unique profiles for some compounds. Hence, bosutinib and sunitinib are ranked 11th and 14th respectively with WS(pIC_50_2) but second and first with WS(pIC_50_1) and WS(pIC_50_0.5). An analysis of the biological profile of sunitinib shows that the highest potency is on the KM12 cell line with a pIC_50_ of 7.4, followed by HOP_92 with a pIC_50_ of 6.4. The presence of only one potency in the 1 and 0.5 log windows for sunitinib explains its excellent rankings for WS(pIC_50_1) and WS(pIC_50_0.5). However, we counted 47 out of 59 potencies in the 2 log window, resulting a poor selectivity score for WS(pIC_50_2). There are excellent correlations of 0.91 and 0.93 between WS(pIC_50_2) and RS(20), and WS(pIC_50_2) and Ssel respectively. WS(pIC_50_1) and WS(pIC_50_0.5) are both well correlated with RS(10), RS(5) and in particular with P_MAX_ (*r* = 0.92 and 0.87). RS(20) shows similar ranking especially with WS(pIC_50_2) and Ssel, and to a lesser extend with RS(10) and P_MAX_. RS(10) and RS(5) present good agreement with most of the metrics except with S(pIC_50_5) and the Gini coefficient. Actually, we observed here a new illustration of the singularity of the Gini coefficient as it is in general poorly correlated with most of the other metrics. Ssel correlates well with WS(pIC_50_2), WS(pIC_50_1), RS(20), RS(10) and P_MAX_. Finally, P_MAX_ is without doubt the metric correlating the most with the other metrics with *r* > 0.5 in all cases except with S(pIC_50_5), S(pIC_50_6) and Gini.

Therefore, the analysis of the twelve metrics on this dataset containing tumor cell lines confirmed our previous observations on the enzymatic dataset. Several metrics seem to be unique in the way of identifying selective compounds. The application of the metrics is highly dependent on the expectations in drug discovery projects and the selectivity profile is usually defined in the TPP.

## Conclusions

The use of selectivity metrics in drug discovery projects is an important parameter to assess the potential safety profile of compounds, especially in the field of kinase research. Several selectivity metrics were presented and discussed using two large in vitro kinase profiling datasets and one cellular screening dataset. Two novel selectivity scores, window score (WS) and ranking score (RS) were developed and compared to existing metrics. A key question was to see whether there might be a clear metric that was definitively better in assessing compound selectivity. Based on our study, currently the answer would appear to be no. Although we have a preference for easily computable and understandable metrics, we think the choice of metric needs to be made carefully. The use of several metrics may help in finding a ranking consensus, but in this case, we suggest using non correlated metrics, like the WS or RS with partition index or selectivity entropy. The use of Gini coefficient on a dataset containing many protein kinases should be taken with caution. As observed in the analysis, its ranking tends to be anomalous and not easy to understand, independent of the dataset.

The standard selectivity score, along with the window score and the ranking score have the advantage of being very easy to compute. Therefore the results they provide are simple to understand and to interpret. Nevertheless, the threshold of the standard selectivity score may be at the origin of some inconsistencies in the results when its value is set too low, as emphasized during our analysis. To avoid this issue, we recommend looking at the activity/affinity range before choosing the threshold. The window score also required a threshold but its impact is less pronounced than that of the standard selectivity score. To differentiate compounds with these metrics, we recommend using at least two different thresholds. A narrow window will identify compounds that bind preferentially one protein kinase, while a large cut-off will identify this same compound if it does not bind many other kinases in the corresponding window. The same conclusion can be drawn for the ranking score. The selected threshold has to represent the activity gap between two inhibited kinases that one considers large enough for compound safety.

To compare two datasets obtained with various techniques returning different activity types, the ranking score is without doubt the metric of choice. As it is based only on the rank determined directly by the data, it does not require any data conversion that could lead to bias.

## Additional file

Additional file 1: Table S1. Ranking of the different metrics computed for the Ambit dataset. **Table S2.** Ranking of the different metrics computed for the Reaction Biology dataset. **Table S3.** Ranking of the different metrics computed for the shared compounds between Ambit dataset (A) and Reaction Biology dataset (B). **Table S4.** Ranking of the different metrics computed for the compounds tested in the NCI-60 assay. (DOCX 184 kb)

## Abbreviations

FDA: Food and Drug Administration
Gini: Gini coefficient
Kd: Dissociation constant
Ki: Constant of inhibition
NCI: US National Cancer Institute
PI: Partition index
RS: Ranking score
S: Standard selectivity score
Ssel: Selectivity entropy
TPP: Target product profile
WS: Window score
