# Supplementary material for: The use of novel selectivity metrics in kinase research
Source: BMC Bioinformatics. 2017 Jan 5;18:17. doi: 10.1186/s12859-016-1413-y (PMC5217660; doi:10.1186/s12859-016-1413-y)
Supplement: Additional file 1: Table S1. — Ranking of the different metrics computed for the Ambit dataset. Table S2. Ranking of the different metrics computed for the Reaction Biology dataset. Table S3. Ranking of the different metrics computed for the shared compounds between Ambit dataset (A) and Reaction Biology dataset (B). Table S4. Ranking of the different metrics computed for the compounds tested in the NCI-60 assay. (DOCX 184 kb) [file 12859_2016_1413_MOESM1_ESM.docx]

**Additional file 1**

**The use of various selectivity scores in kinase research**

Nicolas Bosc^a^, Christophe Meyer^b^ and Pascal Bonnet*,^a^

^a^ *Institut de Chimie Organique et Analytique (ICOA), UMR CNRS-Université d'Orléans 7311, Université d'Orléans BP 6759, 45067 Orléans Cedex 2, France*

^b^ *Centre de Recherche Janssen-Cilag, Campus de Maigremont - CS 10615, 27106 Val de Reuil CEDEX*

^*^Author to whom all correspondence should be addressed:

Pascal Bonnet: Tel: +33 238 417 254, Fax: +33 238 417 254, E-mail: [pascal.bonnet@univ-orleans.fr](mailto:pascal.bonnet@univ-orleans.fr)

**Table S1:** Ranking of the different metrics computed for the Ambit dataset. The first two columns give the Puchem identifier and the common name of the molecules, the twelve other columns give the ranks for each metric. For each ranking column, we colored the cells from red to blue, from the most to the worst selective, respectively. Empty cells represent compounds for which we could not calculate a selectivity value for the given metric.

**Table S2:** Ranking of the different metrics computed for the Reaction Biology dataset. The first two columns give the Puchem identifier and the common name of the molecules, the twelve other columns give the ranks for each metric. For each ranking column, we colored the cells from red to blue, from the most to the worst selective, respectively. Empty cells symbolize compounds for which we could not calculate a selectivity value for the given metric.

**Table S3:** Ranking of the different metrics computed for the shared compounds between Ambit dataset (A) and Reaction Biology dataset (B). The first two columns give the Puchem identifier and the common name of the molecules, the twelve other columns give the ranks for each metric. For each ranking column, we colored the cells from red to blue, from the most to the worst selective, respectively. Empty cells symbolize compounds for which we could not calculate a selectivity value for the given metric.

A.

B.

**Table S4:** Ranking of the different metrics computed for the compounds tested in the NCI-60 assay. The first column gives the common name of the molecules, the twelve other columns give the ranks for each metric. For each ranking column, we colored the cells from red to blue, from the most to the worst selective, respectively. Empty cells symbolize compounds for which we could not calculate a selectivity value for the given metric.
